# Construct validity of the Danish version of the Work Rehabilitation Questionnaire (WORQ)—sensitivity and specificity of the objectively tested physical capacity items

**DOI:** 10.3389/fresc.2023.1115981

**Published:** 2023-04-24

**Authors:** Ida Skovborg Verpe, Katrine Baltzer Thygesen, Reuben Escorpizo, Ole Steen Mortensen, Mette Korshøj

**Affiliations:** ^1^ Department of Occupational and Social Medicine, Holbæk Hospital, Part of Copenhagen University Hospital, Holbæk, Denmark; ^2^ Department of Rehabilitation and Movement Science, College of Nursing and Health Sciences, University of Vermont, Burlington, VT, United States; ^3^ Department of Public Health, Section of Social Medicine, University of Copenhagen, Copenhagen, Denmark; ^4^ The National Research Centre for the Working Environment, Copenhagen, Denmark

**Keywords:** WORQ, rehab assessment, validity, objective measurements, NPV (negative predictive value), PPV (positive predictive value)

## Abstract

**Objective:**

The aim of this study was to examine the construct validity of the Danish version of the Work Rehabilitation Questionnaire (WORQ) and to compare the physical capacity items of WORQ to objective, standardized measures of physical capacity and selected SF-36 physical items.

**Methods:**

The study took place at a job center in Holbæk municipality, and 40 clients of working age were enrolled. Participants completed the interviewer-administered version of WORQ, selected SF-36 items, and underwent objective, physical capacity testing, including a 30-s sit-to-stand-test, a hand-grip-strength test, and a 6-min walk test to estimate cardiorespiratory fitness. Correlations between variables were assessed using Spearman's correlation. Further, cross tabulations and chi-square tests were conducted, and sensitivity, specificity, positive predictive values (PPVs), and negative predictive values (NPVs) were calculated.

**Results:**

We found a moderate to strong correlation between WORQ and SF-36 items and a weak to moderate correlation between physical capacity items of WORQ and objectively tested physical capacity measures. On the basis of cross tabulations, calculations yielded overall higher NPVs than PPVs, whereas sensitivity and specificity varied more, with not one parameter being overall better than the other.

**Conclusion:**

We found evidence of construct validity of the WORQ-Danish. However, our study might also raise a question as to whether objective physical capacity tests are the gold standard for evaluating functioning. Our results are promising, and we suggest further investigations of the screening capabilities of WORQ, alongside other legacy measures or instruments, both self-reported and objective physical measures, to complement information—where specific answers to specific questions trigger work-related actions or interventions

## Introduction

With the advancements made in the fields of medical and technical sciences and the increasing ability to treat and cure diseases, developed nations are facing aging populations and an increase in chronic health conditions, as an increasing number of people are surviving conditions previously considered lethal (1). As a result, rehabilitation is gaining traction as a key health strategy in the 21st century (1). In combination with low birth rates, the workforce demographics are changing; hence, it is essential to optimize work life and increase and sustain workability in the field of occupational health sciences (2).

The challenges of current worker demographics amidst pervasive work disability have driven the need for a more uniform and systematic assessment of functioning and increasing interest in the development of tools for the evaluation of workability, and the World Health Organization (WHO) launched the *International Classification of Functioning, Disability and Health* (ICF) in 2001 (3). The ICF offers a conceptual framework for assessing an individual's functioning, disability, and health based on an integrative biopsychosocial approach. The assessment includes body functions and structures, individual activities and societal participation, and, contextual factors around the environment and the person (4). Within the ICF framework functioning, disability and health can be mapped and internationally classified (4). From a practical relevance and operational point of view as well as in regards to implementation, the ICF core sets were developed (5, 6).

In 2011, Escorpizo et al. developed an ICF-based definition of vocational rehabilitation (VR): “VR is a multi-professional evidence-based approach that is provided in different settings, services, and activities to working age individuals with health-related impairments, limitations, or restrictions with work functioning, and whose primary aim is to optimize work participation” (7).

The identification of the need for an ICF-based instrument to measure work functioning in individuals participating in vocational rehabilitation, and facilitate the multidisciplinary dialogue and communication, spawned the development of the Work Rehabilitation Questionnaire (WORQ) (www.myworq.org) (8). Based on the ICF core sets for vocational rehabilitation, WORQ, as a measurement instrument, represents the practical implementation of a specific set of core sets (8), and it comprises a screening instrument for the mapping of work-related functioning, clinical evaluation, and targeted intervention in vocational rehabilitation (9).

WORQ has been translated and cross-culturally adapted to several other languages and nationalities, including Danish, and it has been well validated by classic psychometric methods (10, 11). However, the WORQ items have never been validated against objective measures of physical capacity, representing a limitation in the validation of WORQ. Traditionally, vocational assessments have striven to provide objective data, and functional capacity evaluations (FCEs) have been used as the gold standard for determining a worker's readiness to return to work. It is a battery of tests that evaluates the musculoskeletal body functions needed to perform work tasks (12). Hence, it is reasonable to evaluate WORQ against objective physical capacity tests assuming that objectively tested physical capacity is the gold standard in assessing work functioning.

This study sought to further investigate the construct validity of WORQ-Danish. Therefore, this paper aims to examine the correlation between (I) WORQ-Danish and SF-36 and (II) the physical capacity items of WORQ and objective measures of physical capacity to elaborate the validity of the WORQ.

We hypothesize that a strong correlation between WORQ-Danish and SF-36 exists, as this relationship has been confirmed in other studies (13). Further, we hypothesize that the physical capacity items of WORQ-Danish will have a strong correlation to the objective, standardized measures of physical capacity; when assuming that objective, functional capacity evaluations are the gold standard for evaluating a worker's functioning.

## Materials and methods

### Design and participants

The study was conducted at a job center in Holbæk municipality. Case managers from the job center recruited working-age clients who are affiliated with the job center for different reasons and are marginalized from the labor market but participating in some kind of vocational rehabilitation (VR).

The study was composed of two parts. One was where participants had to answer the interviewer-administered version of WORQ, administered by the client's case manager, and another was where they completed selected items of the SF-36 questionnaire and underwent physical capacity testing. The physical capacity tests were conducted by a medical student, a specialist in social work and rehabilitation, and a senior researcher. Clients completed the measures in random order, e.g., completed WORQ first and then underwent physical testing or vice versa.

Inclusion criteria were age between 18 and 65 years and competencies in Danish to complete the questionnaires.

Exclusion criteria were (I) having a pacemaker, as it is not possible to measure weight, body mass index (BMI, kg/m^2^), and fat percentage using bioimpedance with such a device implanted; (II) trauma limiting everyday life and any recent operations, as this could potentially impact the physical capacity testing; and (III) pregnancy for ethical reasons.

### Instruments

WORQ-Danish is divided into two parts. Part one contains 17 items on sociodemographic and work-related topics. Part two contains 40 items on social, mental, and physical functioning with a response scale from 0 (no problem) to 10 (complete problem). In addition, two items collect information on (I) time spent per week getting ready in the morning and (II) time spent per week on ongoing treatments (10).

From the 40 items on functioning, we chose the following items to represent the physical capacity items of WORQ: “Overall in the past week, to what extent did you have problems with…” 12: “…keeping your balance while maintaining a position or during movement?”, 14: “…general endurance when performing physical activities?” 15: “…muscle strength?”, 27: “…lifting and carrying objects weighing up to 5 kg?”, 28: “…lifting and carrying objects weighing more than 5 kg?”, 30: “…walking a short distance (less than 1 km)?”, 31: “…walking a long distance (more than 1 km)?”, and 32: “…moving around including crawling, climbing and running?”, 34b: “…riding a bicycle?” and 35: “…getting dressed?”

In addition, the following items were applied: 1: “…not feeling rested and refreshed during the day?” and 37: “…your relationships with people?”.

### SF-36

Participants answered selected items of the SF-36 questionnaire. SF-36 measures health-related quality of life across eight domains (14), and it is thought to be the most widely used general health-status instrument (8).

The 10 SF-36 items regarding physical functioning (the PF scale), 3a–3j, were applied. These items cover physical activity ranging from 3a (vigorous activity) to 3j (bathing or dressing oneself). One item regarding vitality (item 9i) and one regarding social functioning (item 10) were also applied (14).

The participant's answers regarding physical functioning in the 3a–3j items were summed up to a total raw scale score, which can range from 10 to 30. The raw scale score was transformed to a 0–100 scale (transformed scale scores) (15). For analyses regarding physical activity, the transformed scale scores were used.

### Objective physical capacity testing

Participants were exposed to various physical tests designed to capture different facets of physical functioning. Their height (m) was measured not wearing shoes. Weight (kg), fat percentage, and body mass index (BMI, kg/m^2^) were measured by bioelectrical impedance analysis using a Tanita BC-545N (Tanita, Japan). Participants had to empty their pockets, and 1.5 kg of clothing was subtracted from the weight.

Blood pressure and heart rate were measured using an automatic upper arm blood pressure monitor (OMRON HEALTHCARE Co., Japan). Means of three consecutive measures of systolic and diastolic blood pressure, including heart rate on the participants’ left arm, were calculated. Participants were allowed to rest for a minimum of 5 min. They were asked to remain quiet and seated and not to have their legs crossed during measurements.

To assess participants’ muscle strength, their handgrip strength (HGS) was measured using a Jamar Plus+ hand dynamometer (Patterson Medical, MN, USA). The Jamar hand dynamometer is the most widely used tool for assessing HGS and represents the gold standard (16). Measurements were conducted following the “American Society of Hand Therapist” protocol (16), and three consecutive measures on both hands were performed with participants seated, shoulders adducted and neutrally rotated, elbows flexed at 90 degrees, forearm in a neutral position, and feet flat on the floor. A mean was calculated, and analysis was conducted on the mean HGS of the participants’ dominant hand.

Furthermore, participants completed a sit-to-stand test (STST). The STST is considered a measure of lower limb strength, balance, and range of motion (17). A variety of different protocols for STST exist. In this study, the 30-s STST (30sSTST) was chosen and conducted in accordance with the guidelines proposed by Bohannon et al. in 2012 (17). A standard chair with no armrests, stabilized against the wall, was used. Participants were instructed to come forward to the chair and sit down, have their arms folded across their chest, and stand up all the way and sit down again as many times as possible in 30 s.

Finally, to assess participants’ cardiorespiratory capacity, a 6-min walking test (6MWT) was carried out in accordance with the “European Respiratory Society/American Thoracic Society” guidelines (18). The 6MWT provides a simple, valid, and reliable method for assessment of the submaximal, functional exercise capacity of an individual that requires no specialized equipment or highly trained personnel (18, 19). Participants had to walk as many cycles of 60 m as possible for 6 min. The test was conducted on an outdoor, covered pavement. The course was marked at every 5 m, and an orange cone marked the turning point at the end of it. Participants were instructed to walk as fast as possible without running, and they were given time updates and phrases of encouragement at the end of every cycle. Cardiorespiratory fitness (eCRF) (ml O_2_/min/kg) was estimated from their age, sex, and the 6MWT.

### Statistical analysis

Descriptive statistics were performed by calculation of the mean, standard deviation, and range of the background variables, sociodemographic WORQ items (occupational status, educational level, and VR program), physical parameters (age, sex, height, weight, fat percentage, BMI, and blood pressure), and the three tests of physical capacity. Data normality was tested based on histogram analysis and the Kolmogorov–Smirnov test.

The results in the transformed scale score from SF-36 were categorized into two categories (lower or higher than mean) and examined in relation to the relevant physical capacity items of WORQ, which were also categorized into two categories (participants who answered 0–4 and 5–10 on the 11-point scale). On this basis, cross tabulations and chi-square tests were performed.

Relevant WORQ items were correlated to the results of the objective, physical capacity tests and the transformed scale scores of SF-36. Parametric variables were correlated using Pearson's correlation, and nonparametric variables were correlated using Spearman's correlation (20). The WORQ response scale was reversed before calculating correlations to achieve positive correlation coefficients.

Further, the objective, physical capacity measures were also examined in relation to the physical capacity WORQ items through cross tabulations and chi-square tests. To conduct these analyses, the physical parameters were converted from numeric to string variables. The HGS test results were categorized as “below average”, “average”, or “higher than average” based on the norms for average HGS for Danish men and women of working age (21).

The 30sSTST results were categorized by splitting our population data into three equal percentiles, giving us three groups with results ranging from 4 to 11 repetitions, 12 to 16 repetitions, and 17 to 31 repetitions in 30 s. The categorization was conducted using this pragmatic approach due to the lack of norm values for a Danish working-age population without limitations in physical functioning.

The eCRF data were categorized based on norm values into “low”, “medium”, or “high” (22). After data categorization, cross tabulations were conducted in two ways. First, the theoretical cutoff value of the WORQ data was set at 5, and cross tabulations were made between the categorized objective measures and the corresponding WORQ items, categorized into two categories with clients answering from 0 to 4 in one category and from 5 to 10 in another. Afterward, chi-square tests were performed, and sensitivity, specificity, positive predictive values (PPVs), and negative predictive values (NPVs) were calculated based on the results. Second, cross tabulations were made between the categorized objective measures and the raw data of the corresponding WORQ items. This way, a data-driven cutoff value in the WORQ items was established, and cross tabulations, chi-square tests, and sensitivity, specificity, PPV, and NPV calculations were made from these results.

All statistical analyses were performed with software package IBM SPSS Statistics for Mac, version 26.0. The statistical significance level was set at an alpha value of 0.05, two-tailed.

### Ethics

This study was approved by the ethical committee of Region Sjaelland, Denmark, and the Danish data protection agency (journal no. REG-073-2018, file no.: 19-000079/2018-073). Prior to voluntary participation, the participants signed informed consent, and the study was conducted in accordance with the Declaration of Helsinki.

## Results

### Participants

The participants were all clients at a job center in Holbæk municipality. In total, 68 clients were recruited and had appointments with their case managers. However, 28 failed to make an appearance; thus, we collected data from 40 participants, 19 men and 21 women, with a mean age of 42 years (SD = 7.9). None of the participants were working due to either health, ongoing vocational rehabilitation, or other reasons. Everyone had finished primary school, but no one had a university or college degree or any higher level of education. Of 40, 30 participants (72.5%) of our sample were engaged in programs related to preparation for employment such as apprenticeship or internship (Table 1).

**Table 1:** Characteristics of the sample, *n* = 40.

	Mean	SD	Range	*n* [%]
Age	42.40	7.87	28–60	
Sex				
Male				19 [47.5]
Female				21 [52.5]
Weight (kg)	88.37	24.79	54.70–149.50	
Height (m)	1.73	0.10	1.52–1.90	
Fat percentage (*n* = 37)	37.89	9.41	15–54	
Body mass index (kg/m²)	29.55	8.31	16.39–53.63	
Systolic blood pressure (mmHg)	125.49	18.56	100–165	
Diastolic blood pressure (mmHg)	83.98	10.08	64.67–105.67	
Handgrip strength, dominant hand (kg)^a^	33.45	12.39	3.43–56.97	
Sit-to-stand test^b^	15.10	7.18	4–31	
Cardiorespiratory fitness (ml 02/min/kg)^c^	30.89	8.97	10.11–51.41	
Occupational status				
Full-time employed				0 [0]
Part-time employed				0 [0]
On modified or light duty				0 [0]
Not working due to health				14 [35]
Not working due to ongoing vocational rehabilitation				8 [20]
Not working due to other reasons				18 [45]
Educational level				
Not graduated from a primary school				
Primary school				13 [32.5]
High school				8 [20]
Vocationally trained				13 [32.5]
College				6 [15]
University				0 [0]
Postgraduate degree				0 [0]
Vocational rehabilitation program				
Engaged in programs related to preparation for employment such as apprenticeship or internship				29 [72.5]
Engaged in vocational training activities such as in acquiring knowledge and skills for a job, including school training				0 [0]
Engaged in activities to secure or maintain current job				0 [5]
Looking for a (new) job or work				0 [0]
None of the above mentioned				9 [22.5]

^a^ Mean of three consecutive measures.

^b^ Number of repetitions in 30 s.

^c^ Estimated based on age, sex, and result of the 6MWT.

We were not able to retrieve any information on the clients who failed to appear.

### Construct validity of WORQ-Danish with SF-36

Examining the results of the WORQ and SF-36 items distributed in cross tabulations showed a good correlation between participants reporting a low degree of functioning in the transformed scale score of SF-36 and participants reporting a high degree of problem in WORQ (Table 2). The only exception was item 14. Of the 21 participants reporting a high degree of functioning in SF-36 (category 2), 11 answered from 0 to 4 and 10 answered from 5 to 10 in WORQ.

**Table 2:** Cross tabulations between WORQ and SF-36 items, *n* = 40.

WORQ item^b^	SF-36-transformed scale score^a^	
	1 (score < 71.5)	2 (score ≥ .71.5)
14		
0–4	4	11
5–10	15	10
15		
0–4	6	15
5–10	13	6
27		
0–4	8	18
5–10	11	3
28		
0–4	6	16
5–10	13	5
30		
0–4	14	21
5–10	5	0
31		
0–4	6	17
5–10	13	4
32 (*n* = 37)		
0–4	1	13
5–10	16	7
34b (*n* = 35)		
0–4	11	18
5–10	5	1
35		
0–4	16	21
5–10	3	0

^a^ SF-36-transformed scale scores were categorized into two categories: lower or higher than/equal to the mean.

^b^ WORQ items were categorized into two categories: participants answering 0–4 and 5–10 on the 11-point scale.

The established correlations between WORQ-Danish and SF-36 are further represented in the correlation coefficients and chi-square tests (Table 3). The correlation coefficients ranged from 0.405 to 0.738, in other words, from moderate to strong (20). All of the chi-square tests were significant except for one with a marginal *p*-value of 0.058.

**Table 3:** Chi-square tests and correlation analyses between corresponding WORQ and SF-36 items, *n* = 40.

WORQ item^a^	SF-36 item	Spearman's correlation coefficient (*p*-value)	Chi-square test (*p*-value)^d^
14^c^	3a-3j^c^	0.526 (0.000)*	4.18 (0.041)*
15^c^	3a-3j^c^	0.521 (0.001)*	6.35 (0.012)*
27^c^	3a-3j^c^	0.589 (0.000)*	8.34 (0.004)*
28^c^	3a-3j^c^	0.609 (0.000)*	8.02 (0.005)*
30^c^	3a-3j^c^	0.626 (0.000)*	6.32 (0.012)*
31^c^	3a-3j^c^	0.738 (0.000)*	9.95 (0.002)*
32 (*n* = 37)^c^	3a-3j^c^	0.617 (0.000)*	13.65 (0.000)*
34b (*n* = 35)^c^	3a-3j^c^	0.405 (0.016)*	4.13 (0.042)*
35^c^	3a-3j^c^	0.527 (0.000)*	3.59 (0.058)

*Significant results.

^a^ WORQ response scale was reversed before analyses to achieve positive correlations.

^b^ Spearman correlation on the SF-36 raw score (not-transformed scale score) and WORQ raw score.

^c^ Spearman correlation on the raw WORQ score and SF-36-transformed scale score.

^d^ Chi-square tests were performed based on cross tabulations between categorized WORQ scores and categorized SF-36-transformed scale scores (see Table 2).

### Construct validity of WORQ-Danish with objective physical capacity

The results in the objective, physical capacity tests correlating to the corresponding WORQ items on physical capacity varied (Table 4). Item 12 regarding balance was moderately correlated to eCRF but was weakly correlated to the 30sSTST. Item 14 regarding general endurance was weakly correlated to both eCRF and 30sSTST. Item 15 regarding muscle strength was weakly correlated to the 30sSTST but, on the other hand, was moderately correlated to the HGS test. Items 27 and 28 regarding lifting an item weighing more or less than 5 kg were weakly to moderately correlated to the HGS test results. Items 30 and 31 regarding walking a distance of more or less than 1 km also were weakly to moderately correlated to both eCRF and the 30sSTST, although the correlation between item 31 and 30sSTST results was not significant. Item 32 regarding crawling, climbing, and running was moderately correlated to both eCRF and 30sSTST results.

**Table 4:** Spearman correlation analysis between physical capacity WORQ items and objective physical capacity measures, *n* = 40.

	Physical capacity test		
WORQ item^a^	30sSTST	eCRF	HGS
12	0.248 (0.123)	0.445 (0.004)*	
14	0.155 (0.341)	0.264 (0.099)	
15	0.139 (0.393)		0.503 (0.001)*
27			0.328 (0.039)*
28			0.345 (0.029)*
30	0.435 (0.005)*	0.369 (0.019)*	
31	0.302 (0.058)	0.381 (0.015)*	
32	0.498 (0.002)*	0.448 (0.005)*	

*Significant results.

^a^ WORQ response scale was reversed before analysis to achieve positive correlations.

Cross tabulations and chi-square tests were also performed between physical capacity WORQ items and objective measures of physical capacity. As described in the “Materials and methods” section, it was performed both on a theoretical cutoff value in WORQ of 5 and on a data-driven cutoff value in WORQ defined for every single item. The data-driven cutoff values improved the sensitivity and worsened the specificity of the WORQ items. Both PPVs and NPVs improved slightly when using the data-driven cutoff value. Overall, *p* values of the Pearson chi-square tests improved slightly in all tests except for two when using the data-driven cutoff value.

With the data-driven cut-off value, sensitivity ranged from 0.22 to 0.86 and specificity ranged from 0.35 to 0.88, in other words, from poor to excellent (23). PPVs and NPVs ranged from 0.33 to 0.76 and 0.44 to 0.94, respectively, with overall greater NPVs than PPVs (Table 5).

**Table 5:** Chi-square tests and calculations of sensitivity, specificity, positive predictive values (PPVs), and negative predictive values (NPVs) based on cross tabulations on categorized WORQ items and the results of the physical capacity tests, *n* = 40.

WORQ item	Data-driven cutoff value	Physical test	Pearson’s chi-square (*p*)	Sensitivity	Specificity	PPV	NPV
12	3	30sSTST	6.86 (0.032)*	0.57	0.81	0.62	0.78
		eCRF	2.4 (0.3)	0.39	0.76	0.69	0.48
14	5	30sSTST	4.36 (0.113)	0.79	0.35	0.39	0.75
		eCRF	8.6 (0.013)*	0.87	0.53	0.71	0.75
15	4	30sSTST	1.17 (0.557)	0.57	0.38	0.35	0.65
		HGS	3.68 (0.055)	0.86	0.59	0.30	0.94
27	3	HGS	7.03 (0.008)*	0.75	0.75	0.43	0.92
28	4	HGS	3.6 (0.057)	0.75	0.63	0.33	0.91
30	2	eCRF	1.12 (0.57)	0.22	0.82	0.63	0.44
		30sSTST	4.9 (0.086)	0.36	0.88	0.63	0.72
31	4	eCRF	5.5 (0.063)	0.57	0.76	0.76	0.57
		30sSTST	4.45 (0.108)	0.57	0.62	0.44	0.73
32	5	eCRF	4.5 (0.340)	0.76	0.56	0.70	0.64
		30sSTST	7.2 (0.125)	0.85	0.50	0.48	0.86

*Significant results.

## Discussion

This study shows an overall moderate to strong correlation between WORQ-Danish and SF-36 items and, further, a weak to moderate correlation between the objective, physical capacity measures and the physical capacity items of WORQ-Danish. Our sensitivity/specificity and PPV/NPV analyses yielded overall best results for the NPVs. Hence, WORQ is best at identifying participants who do not have a problem with a given task.

The correlations between the 30sSTST and the physical capacity WORQ items varied from 0.139 for item 14 (muscle strength) to 0.498 for item 32 (crawling, climbing, and running), and most of the correlations were weak. This raises some questions regarding the 30sSTST, which is a test developed to measure mobility among elderly people (17, 24). Indeed, we were not able to find norm values for people of working age with no limitation in physical capacity. As so, it is reasonable to believe that the 30sSTST is not sensitive enough to measure the limitations in the physical capacity that our sample may experience since they are adults of working age and therefore in overall better shape than the older adults this test was intended for.

The correlations between the results for the eCRF and the selected WORQ items varied from 0.264 for item 14 (general endurance) to 0.448 for item 32 (crawling, climbing, and running), with most of the correlations being of moderate strength. This supports the fact that completing a 6MWT depends on both balance and mobility. The fact that it was weakly correlated to the general endurance item might say more about our sample than the WORQ item itself; see later in the discussion.

Finally, the correlations between the results for the HGS and the selected WORQ-items varied from 0.328 for item 27 (lifting objects weighing 5 kg or less) to 0.503 for item 15 (muscle strength), thus overall moderate correlations. Compared to the other two tests, testing for handgrip strength using a dynamometer is a relatively simple test that isolates handgrip strength and is not dependent on other factors such as balance, muscle strength in lower extremities, and cardiorespiratory fitness. This could be one explanation as to why results are better for this test than the other two measures of physical capacity.

The results should also consider item 12 (balance) and 14 (general endurance) in WORQ. Balance is a rather complex concept that even healthcare providers sometimes use with no clear definition in mind (25). When asking clients or patients to self-evaluate problems with balance, it is pertinent to ask whether they have the same understanding of the concept as we do.

Item 14 regarding general endurance was weakly correlated to both eCRF and 30sSTST.

Overall, these results should be seen in the light of the characteristics of the included population in this study. These participants are not assumed to perform high levels of leisure time physical activity (26); therefore, when answering questions about their physical capacity, they might tend to underestimate it or simply do not know how much they are capable of. This uncertainty regarding item 14 is further reflected in the cross tabulation between WORQ and SF-36 items. The table shows that 15 of 19 participants reporting a low degree of functioning in SF-36 also reported a high degree of problem with general endurance in WORQ. On the contrary, 11 of 21 participants reporting a high degree of functioning in SF-36 reported a high degree of problem with general endurance in WORQ. It seems that item 14 of WORQ cannot separate those who have a high degree of general endurance from those who do not. This might reflect that general endurance when performing a physical activity depends not only on one's objective, physical capacity but also on one's state of mind and personal factors. Hence, it underlines the importance of the biopsychosocial approach in the ICF and the WORQ questionnaire, and it supports the fact that when working in vocational rehabilitation, unidimensional evaluations like the functional capacity evaluations (FCEs) are not capable of capturing the multifaceted process of return to work (12). In this line of work, both measures of physical capacity and personal, social, and environmental evaluations are of greatest interest to fully understand a person's workability.

We chose to perform cross tabulations and chi-square tests under the presumption that the WORQ is a screening tool for the assessment of work functioning and therefore it would be of value to calculate sensitivity, specificity, PPV, and NPV. When dealing with these parameters, it is important to keep in mind that a tradeoff between the parameters often exists. You will often see a great sensitivity at the expense of the specificity of the test and vice versa and likewise with the PPV and NPV. In this study, results showed overall greater NPVs than PPVs. Sensitivity and specificity varied, with not one of them being overall better than the other. A greater NPV on behalf of PPV means that the questionnaire is better at finding those who do not have a given problem or impairment than those that do have a given problem. This means that when participants answer “no degree of problem” in WORQ, this is likely to be true. On the other hand, there is a greater insecurity in the group of participants answering “high degree of problem” as to whether they really have the given problem or impairment or not, keeping in mind that only the purely physical aspects of the concepts were examined.

Our results altered a bit when, instead of a theoretical cutoff value of 5 in the WORQ data, we set a more data-driven cutoff value by looking at the data before defining the cutoff value. It follows that when using the WORQ as a screening tool, one might need to have different cutoff values for different items, at least to create the greatest possible balance between sensitivity and specificity and PPV and NPV. However, this effect is also affected by our small sample size, and it is reasonable to assume that differences would diminish with a larger sample size and more heterogeneity.

These preliminary results provide the evidence of construct validity needed to use WORQ-Danish for assessing work functioning. The validity of WORQ has been examined in several other studies (9, 13); however, to the best of our knowledge, this is the first time it has been investigated by comparing WORQ with objective, physical capacity measures. In this study, we tested it on 40 working-age clients at a job center in Holbæk municipality, but WORQ has the potential to be used in a wide variety of clinical settings for a quick and systematic assessment of work functioning. Our results are promising, and we suggest further investigations of the screening capabilities of WORQ, alongside other legacy measures or instruments, both self-reported and objective physical measures, to complement information—where specific answers to specific questions trigger work-related actions or interventions.

### Methodological considerations

Overall, our validation efforts showed promising results. However, this study had a significant limitation in that the study population was no larger than 40 participants, representing a moderate sample size, when using the COSMIN checklist (27). Also, participants were from a job center at Holbæk municipality and, therefore, represent a homogeneous and highly selective population, leading to a potential sample bias. Thus, results can only be used to conclude on behalf of our sample and should not be generalized to the entire population of Denmark. To generalize results, we would need to replicate our study in a larger and more diverse population.

Another consequence of the small sample size was a low degree of significant results when performing chi-square tests on the physical capacity measures, and also, many of the cells in the expected count had values less than 5. This, of course, decreases the quality of the results. However, this does not prevent us from detecting trends and does not affect the calculation of sensitivity, specificity, PPV, and NPV from the results in the cross tabulations.

As mentioned earlier, to the best of our knowledge, this is the first time WORQ has been validated against objective, physical capacity measures. Although some degree of correlation was found, our results also showed that one's self-reported degree of functioning and workability might not have a strictly linear relationship with objective, physical capacity measures. This reflects that functioning and workability are multifaceted concepts, depending not only on purely physical performance and bodily structures but also on environmental and personal factors, and it underlines the importance of WORQ's foundation in the ICF model.

These preliminary results call for further research to confirm and follow up on our results.

## Conclusion

This study represents the preliminary results regarding the construct validity of the WORQ-Danish. We found evidence that WORQ-Danish is moderate to highly correlated to the SF-36 questionnaire. Results from the correlation to the physical capacity measures varied, but overall, we saw a weak to moderate correlation. Our results further highlighted the importance of a biopsychosocial approach in vocational rehabilitation.

Regarding the use of WORQ as a screening tool, our results showed that the questionnaire is better at finding those who do not have a given problem or impairment than those who do when only considering the physical aspects of the problem or impairment.

Our study had some clear methodological limitations. However, it provides a sound basis for further research on the psychometric properties of WORQ to address work disability and to help guide vocational rehabilitation efforts.

## Data Availability

The original contributions presented in the study are included in the article/Supplementary Material, further inquiries can be directed to the corresponding author.
